# Galantamine plasma concentration and cognitive response in Alzheimer’s disease

**DOI:** 10.7717/peerj.6887

**Published:** 2019-05-02

**Authors:** Yi-Ting Lin, Mei-Chuan Chou, Shyh-Jong Wu, Yuan-Han Yang

**Affiliations:** 1 Department of Family Medicine, Kaohsiung Medical University Hospital, Kaohsiung Medical University, Kaohsiung, Taiwan, Taiwan; 2 Department of Medical Sciences, Molecular Epidemiology, Uppsala Universitet, Uppsala, Uppsala, Sweden; 3 Department of Neurology, Kaohsiung Medical University Hospital, Kaohsiung Medical University, Kaohsiung, Taiwan, Taiwan; 4 Department of Neurology, Kaohsiung Municipal Ta-Tung Hospital, Kaohsiung Medical University, Kaohsiung, Taiwan, Taiwan; 5 Department of Medical Laboratory Science and Biotechnology, Kaohsiung Medical University, Kaohsiung, Taiwan, Taiwan; 6 Department of and Master’s Program in Neurology, Faculty of Medicine, Kaohsiung Medical University, Kaohsiung, Taiwan, Taiwan; 7 Neuroscience Research Center, Kaohsiung Medical University, Kaohsiung, Taiwan

**Keywords:** Alzheimer’s disease, Galantamine, Cognitive response, Cholinesterase inhibitors, MMSE

## Abstract

**Background:**

Galantamine has been approved for the treatment of Alzheimer’s disease (AD). However, there are few studies which have reported the association between cognitive responses and galantamine plasma concentration. The aim of this study was to determine the correlation between galantamine plasma concentration and the subsequent cognitive response following treatment in AD patients.

**Methods:**

AD sufferers who continuously took 8 mg/d galantamine for at least 6 months without previous exposure to other kinds of AChEI such as donepezil, rivastigmine, or memantine were included in this cohort study. The assessments included the Mini Mental Status Examination (MMSE), Clinical Dementia Rating Scale (CDR) and the Cognitive Assessment Screening Instrument (CASI). Each subdomain of the CASI assessment was conducted at baseline and after 6 months of galantamine. The plasma concentrations of galantamine were measured by capillary electrophoresis after 6 months of the treatment. Logistic regression was performed to adjust for age, gender, apolipoprotein E ε4 genotype status, and baseline score to investigate the association between galantamine plasma concentrations and the cognitive response.

**Results:**

The total sample consisted of 33 clinically diagnosed AD patients taking galantamine 8 mg/d for 6 months. There was no linear correlation between galantamine concentration and cognitive response in patients. However, 22 patients were responsive to the treatment in the long-term memory domain. In CASI subset domain, concentration improved during the 6 months follow up.

**Conclusions:**

In the limited samples study, galantamine mostly benefitted the cognitive domain of long-term memory. The benefits were not related to the galantamine plasma concentration. Objective intra-individual evaluation of therapeutic response should be encouraged.

## Introduction

Galantamine is one of the acetylcholinesterase inhibitors (AChEIs) that have been approved as the main treatment for mild to moderate Alzheimer’s disease (AD) (Lanctot et al., 2003; Pirttila et al., 2004; Wilcock, Lilienfeld & Gaens, 2000), which can inhibit enzymes from degrading acetylcholine. Acetylcholinesterase inhibitor can slow the decline of cognitive function in patients with AD (Lilienfeld, 2002; Scott & Goa, 2000). Various dosages of galantamine have been proposed to provide the therapeutic benefits for AD (Wilkinson & Murray, 2001). However, the response ratio has varied by individuals and baseline characteristics (Bickel et al., 1991; MacGowan, Wilcock & Scott, 1998; Zhao et al., 2002). Previous studies have stated that several factors influence the treatment outcome, including sex, body weight, neuroanatomical characteristics, baseline cognitive function, gene polymorphism, cytochrome P450 and apolipoprotein E (ApoE) (Cacabelos et al., 2007; Chen & Hu, 2006; Geerts et al., 2005; MacGowan, Wilcock & Scott, 1998). The meta-analysis article showed that better cognitive outcome was related to higher dosages of AChEI treatment (Lanctot et al., 2003). Only one article in Sweden demonstrated that higher galantamine plasma concentration was positively correlated to higher dosages of galantamine intake, but no relationship was found between the concentration of galantamine and positive short-term cognitive outcome from the treatment (Wattmo et al., 2013). It is still to be determined whether higher galantamine plasma concentration is related to better therapeutic response—especially in Asia where, to our knowledge, no study has investigated the relationship between cognitive response and the plasma concentration of galantamine in AD patients.

In order to reflect and examine the therapeutic response of galantamine in AD patients from Taiwan, we have traced the change of psychometrics of AD patients in relation to the plasma concentration of galantamine to evaluate the cognitive response and clinical outcomes of AD patients treated with galantamine.

## Materials & Methods

### Patients

All patients were recruited from the Neurological Department of Kaohsiung Medical University Hospital, a medical center in southern Taiwan. Data were collected as previously described (Yang et al., 2013). Specifically, patients with AD who continuously took galantamine 8 mg/d for at least 6 months without previous exposure to any kind of AChEI such as donepezil, rivastigmine, or memantine were included in this study. Patients with other conditions possibly contributing to the diagnosis of AD were excluded, such as hypothyroidism, vitamin B12 and folic acid deficiency, hypercalcemia, neurosyphilis, HIV infection, and cerebrovascular disease. All of the primary outcomes of the participants were measured during their first 6-month follow-up visit after starting the galantamine 8 mg/d treatment. All procedures were approved by the Kaohsiung Medical University Hospital Institutional Review Board (KMUH-IRB-970049 and KMUH-IRB-990301), and written informed consent was obtained from all participants or their legal representatives.

### Evaluation

A series of neuropsychological assessments were conducted twice, once in the beginning before the administration of the medication and again roughly 6 months after treatment to evaluate the therapeutic response. The neuropsychological assessments, including the Mini Mental Status Examination (MMSE) (Folstein, Folstein & McHugh, 1975), Cognitive Assessment Screening Instrument (CASI) (Teng et al., 1994) and Clinical Dementia Rating Scale (CDR) (Morris, 1993), were conducted by a senior neuropsychologist and an experienced physician based on information from a knowledgeable collateral source (usually a spouse or adult child). The assessments were also mentioned before (Yang et al., 2013).

The CASI is an objective test that can be administered in approximately 15 to 20 min. The 25 subdomains of the test contribute to the subscales of the CASI assessment, used to evaluate the nine cognitive domains of attention, concentration, orientation, short-term memory, long-term memory, language abilities, visual construction, category fluency, and abstraction and judgment. The maximum scores for the subscales range from 8 to 18, with a total score of 100 (Teng et al., 1994). A cognitive response after therapy was defined by the intra-individual comparisons of the differences of the MMSE (Folstein, Folstein & McHugh, 1975), CDR-SB (Morris, 1993), and the total score from each subscale of the CASI assessment (Teng et al., 1994)—before and after administration of galantamine. If an individual’s second CASI subscale score was higher than or equal to the first, it was considered to be a better response in the cognitive domain. Otherwise, it was considered a poor response. The same algorithm was also applied to MMSE. Compared to first evaluation, higher or equal MMSE score in second evaluation would be considered as better response.

The diagnosis of AD was based on the criteria stated by The National Institute of Neurological and Communicative Disorders and Stroke, as well as The Alzheimer’s Disease and Related Disorders Association, referring to a series of comprehensive neuropsychological tests, including the MMSE (Folstein, Folstein & McHugh, 1975), CASI (Teng et al., 1994), Neuropsychiatric Inventory, and CDR scale (Morris, 1993). The CDR scale is a global scale to rate cognitive performance in six domains: memory, orientation, judgment and problem solving, community affairs, home and hobbies, and personal care through interviews with the patients. During the diagnostic work up, MRI or CT and a series of comprehensive neuropsychological test were done. Meanwhile, the factors contributing to cognitive staus were excluded in otder to have the accurate diagnosis of AD.

### Apolipoprotein E genotyping

For every AD patient, restriction enzyme isotyping of the ApoE allele was performed following a modification of the protocol developed by Pyrosequencing (http://www.pyrosequencing.com) and has been described previously (Yang et al., 2013).

An amount of 10 ng of DNA was amplified in a 20 HL reaction volume, in which deoxyguanosine triphosphate was replaced by a mixture of 25% deoxyguanosine triphosphate and 75% deoxyinosine triphosphate to facilitate analysis of the GC-rich fragment. A 276-bp fragment was generated using the forward primer AGA CGC GGG CAC GGC TGT and the reverse biotin-labeled primer CTC GCG GAT GGC GCT GAG. Single-strand DNA, prepared using streptavidin-coated beads and the APoE gene variants at codons 112 and 158, were pyrosequenced using the following primers and dispensation order: SNP112 GAC ATG GAG GAC GTG and SNP158 CCG ATG ACC TGC AGA and dispensation order GCTGAG CTAGCGT. Individuals with more than one copy of the ApoE ε4 allele were considered ε4 positive.

### Plasma concentration of galantamine

Every patient was treated continuously with galantamine for at least 6 months. Blood samples were collected from these patients during specific visits and the plasma concentration of galantamine was considered to be at a steady state for our recruited patients.

The plasma concentration of galantamine was measured through capillary electrophoresis, with a Beckman P/ACEMDQ system (Beckman Coulter, Fullerton, CA) equipped with a UV detector and a liquid-cooling device. A detailed description of the methods utilized in measuring the plasma concentration of galantamine has been published elsewhere (Hsieh et al., 2009).

### Statistical analysis

Independent t-tests for the two independent groups were used to assess the differences between groups of herapeutic response improved or worsened in CASI subdomain and CDR score. Paired T-tests was also applied to compare the individual difference between first and second evaluations. The total CASI assessment scores were assessed with an interval of more than 6 months. Multiple logistic regression models were used to calculate odds ratios (ORs) and 95% confidence intervals (CIs) for the association between the cognitive response and plasma concentration of galantamine. This model was adjusted for age, sex, education, baseline MMSE, baseline CDR scores and ApoE ε4 status. The dependent variable in each logistic regression model was either a better response or a poor response. The independent variables, which were age, education and the plasma concentration of galantamine, were treated as continuous variables. This was done by 1-year increments for age and education and 1 ng/mL increments for the plasma concentration of galantamine–in contrast to sex and ApoE ε4 status, which were treated as dichotomous categorical variables. All analyses were performed using the SAS statistical software (version 9.2; SAS Institute Inc., Carey, NC, USA). Bonferroni correction was applied to adjust the model for multiple comparisons and an adjusted *p* < 0.0045 was considered statistically significant.

## Results

Thirty-three patients with AD, with CDR 0.5 to 2.0 throughout the two assessments, were included in our statistical analysis. Among them, 21 (60%) were female and 13 patients (39.4%) were ApoE ε4 carriers. The average age of the patients was 78.3 years old, with an average of 5.4 years spent in education. The mean baseline scores of the CDR-SB, MMSE and CASI were 5.0, 16.8, and 60.6 respectively. The second evaluation of MMSE and CDR-SB were 15.2 ± 7.0 and 5.7 ± 3.2. The mean (± SD) plasma concentration of galantamine was 83.7 ± 70.3 ng/mL (Table 1). The analysis of the therapeutic response of each of the CASI assessment subdomains revealed a significant decline in the concentration domain but non-significant decrease in the other CASI assessment scores. The total score from the second CASI assessment showed a significant decrease of 5.6 ± 15.1 (*p* = 0.042, Table 2).

**Table 1:** Demographic characteristics of recruited subjects.

	Total Patient No. = 33		
Age, years (mean ± SD)	78.3 ± 6.7		
Gender [patient No., (%)]	
Male	13 (39%)		
Female	20 (61%)		
Education, years (mean ± SD)	5.4 ± 4.3		
ApoE ε4(+) [patient No., (%)]	13 (39.4%)		
CDR evaluation before and after galantamine used	1st evaluation	2nd evaluation	*p*-value
CDR [patient No., (%)]			<0.001
0.5	15 (45.5)	10 (30.3)	
1.0	13 (39.4)	17 (51.5)	
2.0	5 (15.2)	5 (15.2)	
3.0	0 (0)	1 (3.0)	
CDR-SB (mean ± SD)	5.0 ± 3.2	5.7 ± 3.2	0.089
MMSE (mean ± SD)	16.8 ± 6.8	15.2 ± 7.0	0.056
Galantamine (mean ± SD) ng/ml		83.7 ± 70.3	

**Notes.**

ApoE apolipoprotein E CDR Clinical Dementia Rating CDR-SB Commercial Dispute Resolution sum of boxes MMSE mini–mental state examination SD Standard Difference

**Table 2:** Baseline and follow-up assessment with CASI at 6 months (mean CASI total and subdomain scores).

Total patient No. = 33				
Cognitive domain	1st evaluation (mean ± SD)	2nd evaluation (mean ± SD)	Mean difference (mean ± SD)	*p*-value
Attention (0–8)	5.7 ± 2.1	5.0 ± 2.3	−0.7 ± 2.1	0.080
Concentration (0–10)	5.6 ± 3.4	4.3 ± 3.8	−1.3 ± 3.0	0.022^*^
Orientation (0–18)	11.2 ± 5.5	10.1 ± 5.7	−1.1 ± 4.3	0.157
Short-term memory (0–12)	3.3 ± 3.5	2.7 ± 3.2	−0.5 ± 1.9	0.135
Long-term memory (0–10)	8.6 ± 2.2	8.0 ± 3.0	−0.7 ± 2.3	0.112
Language abilities (0–10)	7.0 ± 2.4	6.6 ± 2.6	−0.5 ± 2.0	0.194
Visual construction (0–10)	7.3 ± 3.4	7.0 ± 3.1	−0.4 ± 3.3	0.530
Category fluency (0–10)	4.4 ± 2.3	4.4 ± 2.5	−8.8 ± 4.3	1.000
Abstraction and judgment (0–12)	5.9 ± 2.9	5.8 ± 3.4	−0.1 ± 3.0	0.865
CASI total (0–100)	60.6 ± 22.3	55.0 ± 23.8	−5.6 ± 15.1	0.042^*^

**Notes.**

CASI The Cognitive Abilities Screening Instrument SD Standard Difference

* *p*-value < 0.05.

**Figure 1:**
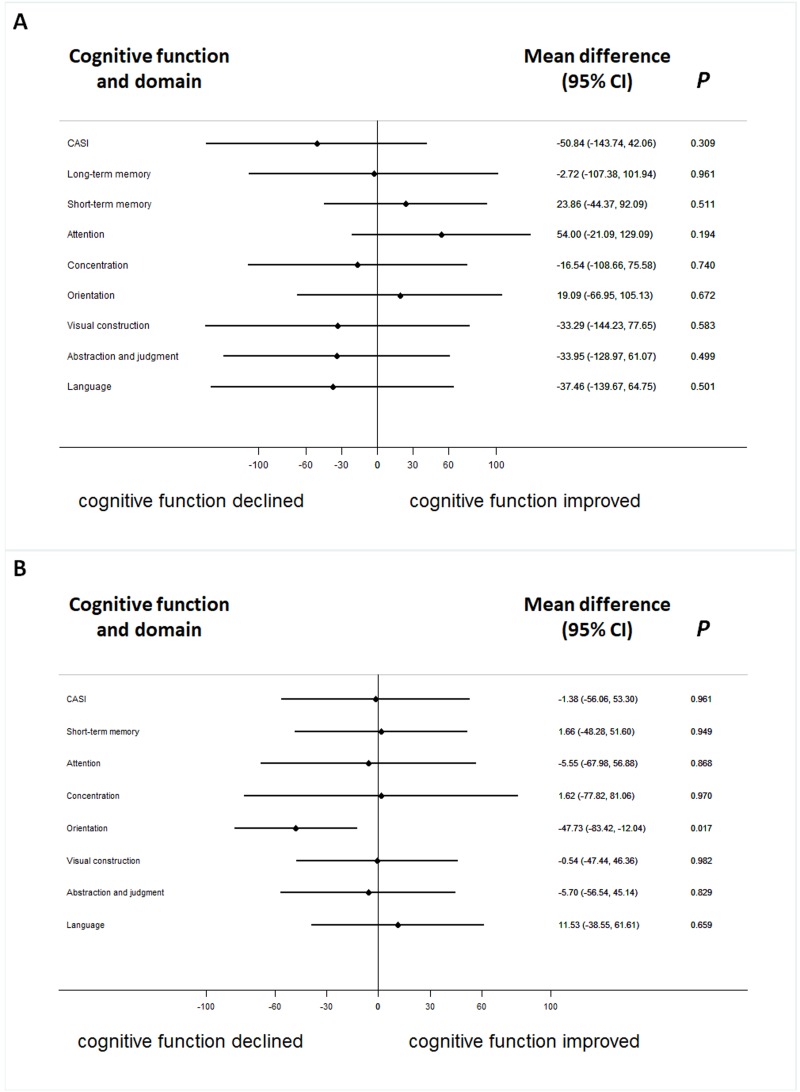
The difference of serum galantamine concentration between improved and worsened group in patients with or without ApoE ε4(+). The difference of serum galantamine concentration between improved and worsened groups in patients with ApoE ε4(+) (A) or without (B).

In therapeutic response as evaluated by intra-individual comparisons, which means to compare cognitive performance before and after the treatment per patient, thirteen (39.4%) patients were responsive to the treatment according to total score from the CASI assessment. Long-term memory had the highest response ratio (66.7%) in contrast to category fluency, which had the lowest therapeutic response ratio in the nine cognitive domains of the CASI assessment (Table 3). The difference of CASI assessment between improved and worsened group were both insiginificant by stratifying ApoE ε4(+) (Fig. 1).

**Table 3:** Therapeutic response of Alzheimer’s disease treated with galantamine.

Total patient No. (*N* = 33)	Therapeutic response improved^a^			Therapeutic response worsened^b^			
Cognitive domain	Patient No.	%	galantamine concentration (ng/ml) (mean ± SD)	Patient No.	%	galantamine concentration (ng/ml) (mean ± SD)	*p*-value
Attention	18	54.6	79.4 ± 72.9	15	45.5	89.0 ± 69.2	0.702
Concentration	18	54.6	89.7 ± 67.2	15	45.5	76.5 ± 75.5	0.600
Orientation	16	48.5	109.2 ± 71.2	17	51.5	59.7 ± 62.3	0.041
Short-term memory	17	51.5	88.9 ± 78.3	16	48.5	78.3 ± 72.9	0.673
Long-term memory	22	66.7	86.6 ± 70.9	11	33.3	77.9 ± 72.2	0.744
Language abilities	18	54.6	74.6 ± 59.3	15	45.5	94.7 ± 82.5	0.423
Visual construction	18	54.6	81.6 ± 63.0	15	45.5	86.2 ± 80.4	0.854
Category fluency	0	0	0	33	100	83.7 ± 70.3	–
Abstraction and judgment	19	57.6	86.5 ± 77.6	14	42.4	80.0 ± 61.7	0.800
CASI total	13	39.4	100.5 ± 69.1	20	60.6	72.8 ± 70.7	0.275
CDR-SB	16	48.5	103.5 ± 79.3	17	51.5	65.1 ± 56.8	0.118

**Notes.**

CASI The Cognitive Abilities Screening Instrument CDR-SB Commercial Dispute Resolution sum of boxes SD Standard Difference

a Difference of two tested scores ≥ 0.

b Difference of two tested scores <0.

**Figure 2:**
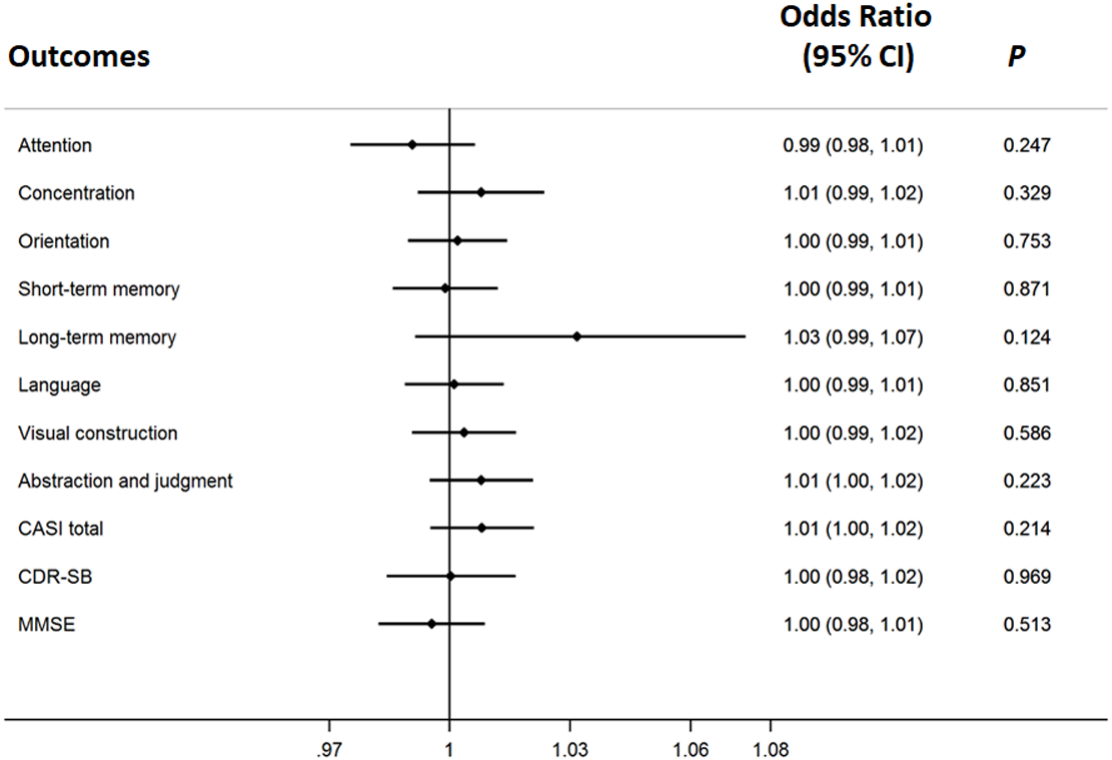
The therapeutic response of galantamine concentration in Alzheimer’s disease. The adjusted effects of the concentration of galantamine in each cognitive domain to individualized therapeutic response in Alzheimer’s disease.

The cognitive responses, measured by the CASI assessment with its nine cognitive domains, were not significantly associated with the concentration of galantamine after adjusting for age, gender, ApoE ε4, education and baseline cognitive performance in the logistic regression model with Bonferroni corrections (Fig. 2). ApoE genome typing was not related to the therapeutic response in each domain. The cognitive response evaluation of the total score for the CASI assessment was 0.992 (0.979–1.006) (Fig. 2). The OR for the CDR-SB was 0.888 (0.620–1.272) after galantamine treatment. The change in cognitive function assessed by the MMSE was also non-significant.

## Discussion

In this study, we found that the long-term memory domain in patients will benefit most from galantamine treatment (66.7%) in contrast to the categorical fluency domain that was not responsive to galantamine treatment. These findings were, in part, similar for donepezil treatment (Yang et al., 2013). However, this was not true for rivastigmine treatment, where the CASI domain that benefited the most from the treatment was categorical fluency (Chou et al., 2012).

The cognitive therapeutic response was similar in donepezil and galantamine treatment because both drugs are acetyl-cholinesterase inhibitors (Ritchie et al., 2004). Rivastigmine functions as both an acetyl-cholinesterase and a butyl-cholinesterase inhibitor (Polinsky, 1998; Ritchie et al., 2004), which may be why a different cognitive response was observed for this treatment. Furthermore, donepezil and galantamine metabolism involves the use of similar hepatic enzymes (Nordberg & Svensson, 1998), thus they have similar serum concentrations to the cognitive responses.

When the therapeutic responses were examined in evaluating the change of group-mean, we found that CASI total score with each of its individual sub-items, MMSE, and CDR-SB, did not improve throughout our observational period. Actually it could be treated as a disease deterioration progressively. However, if the therapeutic response was examined individually, for intra-individual comparisons, the overall response ratio was 48.5% to 66.7% (Table 3). Such findings also highlighted the importance of intra-individual comparisons that will be more practicable in clinical settings because in the clinic we treat an individual, not a group, and evaluate the therapeutic response of an individual, not a group.

In evaluating the effects of galantamine on the therapeutic responses, we found that the serum concentration of galantamine was not significantly associated with the therapeutic response. Previous studies mostly focused on the correlation between dosage of galantamine taken and the therapeutic response. The effect on therapeutic response should be discussed by dosage effect or blood concentration effect. According to previous reports, better cognitive function improvement when patients were under a higher dosage of galantamine treatment (Wilkinson & Murray, 2001; Raskind et al., 2000; Tariot et al., 2000; Wilkinson & Murray, 2001). However, there was a study revealed that no relationship was found between concentration and short-term cognitive treatment response (Wattmo et al., 2013). The same result was found in the present study. However, the 8 mg dose/day administered in the current study might not be able to reach the optimal level of serum galantamine concentration to achieve maximal effect on therapeutic response. Higher doses would be considered for the future study. Meanwhile, Previous studies did not examine the change during individualized treatment, but in group means, and used different psychometrics that would have different therapeutic outcomes. Such study designs could not reflect the real-world condition in which we treat a patient on an individualized basis, not in a group.

This study is important to compare the different cognitive responses in various neuropsychological domains with respect to different plasma concentrations of galantamine. Intra-individual comparison was also conducted. Intra-individual examination is better than comparing the change of group-mean as a therapeutic response because we are treating a patient not a group (Chou et al., 2012; Yang et al., 2013). Intra-individual differences could influence therapeutic response of AchEI inhibitors, such as sex, age, cognitive status, education level (Haywood & Mukaetova-Ladinska, 2006; Lopez et al., 2002). In the present study, patients with low education level and low cognitive status at baseline could contribute to unfavorable therapeutic response to galantamine.

## Conclusions

This pilot cohort study indicated the most beneficial cognitive domain after treating galantamine was long-term memory, but category fluency had no response to galantamine. Galantamine plasma concentration had no significant association with the treatment response in each domain for evaluation. In order to have better therapeutic response and have less side effects from galantamine, adequate dosage of galantamine in the treatment of AD should be considered.

##  Supplemental Information

10.7717/peerj.6887/supp-1 Dataset S1 Raw data Raw data that the patients were willing to share publicly.

## Additional Information and Declarations

### Data Availability

The following information was supplied regarding data availability: The raw measurements are available in File S1.

## References

[ref-1] Bickel U, Thomsen T, Fischer JP, Weber W, Kewitz H (1991). Galanthamine: pharmacokinetics, tissue distribution and cholinesterase inhibition in brain of mice. Neuropharmacology.

[ref-2] Cacabelos R, Llovo R, Fraile C, Fernandez-Novoa L (2007). Pharmacogenetic aspects of therapy with cholinesterase inhibitors: the role of CYP2D6 in Alzheimer’s disease pharmacogenetics. Current Alzheimer Research.

[ref-3] Chen HH, Hu CJ (2006). Genetic characteristics of dementia in Taiwan. Acta Neurologica Taiwanica.

[ref-4] Chou MC, Chen CH, Liu CK, Chen SH, Wu SJ, Yang YH (2012). Concentrations of rivastigmine and NAP 226-90 and the cognitive response in Taiwanese Alzheimer’s disease patients. Journal of Alzheimer’s Disease.

[ref-5] Folstein MF, Folstein SE, McHugh PR (1975). “Mini-mental state”. A practical method for grading the cognitive state of patients for the clinician. Journal of Psychiatric Research.

[ref-6] Geerts H, Guillaumat PO, Grantham C, Bode W, Anciaux K, Sachak S (2005). Brain levels and acetylcholinesterase inhibition with galantamine and donepezil in rats, mice, and rabbits. Brain Research.

[ref-7] Haywood WM, Mukaetova-Ladinska EB (2006). Sex influences on cholinesterase inhibitor treatment in elderly individuals with Alzheimer’s disease. The American Journal of Geriatric Pharmacotherapy.

[ref-8] Hsieh YH, Yang YH, Yeh HH, Lin PC, Chen SH (2009). Simultaneous determination of galantamine, rivastigmine and NAP 226-90 in plasma by MEKC and its application in Alzheimer’s disease. Electrophoresis.

[ref-9] Lanctot KL, Herrmann N, Yau KK, Khan LR, Liu BA, LouLou MM, Einarson TR (2003). Efficacy and safety of cholinesterase inhibitors in Alzheimer’s disease: a meta-analysis. Canadian Medical Association Journal/Journal de l’Association Medicale Canadienne.

[ref-10] Lilienfeld S (2002). Galantamine—a novel cholinergic drug with a unique dual mode of action for the treatment of patients with Alzheimer’s disease. CNS Drug Reviews.

[ref-11] Lopez OL, Becker JT, Wisniewski S, Saxton J, Kaufer DI, DeKosky ST (2002). Cholinesterase inhibitor treatment alters the natural history of Alzheimer’s disease. Journal of Neurology, Neurosurgery and Psychiatry.

[ref-12] MacGowan SH, Wilcock GK, Scott M (1998). Effect of gender and apolipoprotein E genotype on response to anticholinesterase therapy in Alzheimer’s disease. International Journal of Geriatric Psychiatry.

[ref-13] Morris JC (1993). The Clinical Dementia Rating (CDR): current version and scoring rules. Neurology.

[ref-14] Nordberg A, Svensson AL (1998). Cholinesterase inhibitors in the treatment of Alzheimer’s disease: a comparison of tolerability and pharmacology. Drug Safety.

[ref-15] Pirttila T, Wilcock G, Truyen L, Damaraju CV (2004). Long-term efficacy and safety of galantamine in patients with mild-to-moderate Alzheimer’s disease: multicenter trial. European Journal of Neurology.

[ref-16] Polinsky RJ (1998). Clinical pharmacology of rivastigmine: a new-generation acetylcholinesterase inhibitor for the treatment of Alzheimer’s disease. Clinical Therapeutics.

[ref-17] Raskind MA, Peskind ER, Wessel T, Yuan W (2000). Galantamine in AD: a 6-month randomized, placebo-controlled trial with a 6-month extension. The Galantamine USA-1 Study Group. Neurology.

[ref-18] Ritchie CW, Ames D, Clayton T, Lai R (2004). Metaanalysis of randomized trials of the efficacy and safety of donepezil, galantamine, and rivastigmine for the treatment of Alzheimer disease. The American Journal of Geriatric Psychiatry.

[ref-19] Scott LJ, Goa KL (2000). Galantamine: a review of its use in Alzheimer’s disease. Drugs.

[ref-20] Tariot PN, Solomon PR, Morris JC, Kershaw P, Lilienfeld S, Ding C (2000). A 5-month, randomized, placebo-controlled trial of galantamine in AD. The Galantamine USA-10 Study Group. Neurology.

[ref-21] Teng EL, Hasegawa K, Homma A, Imai Y, Larson E, Graves A, Sugimoto K, Yamaguchi T, Sasaki H, Chiu D, White LR (1994). The Cognitive Abilities Screening Instrument (CASI): a practical test for cross-cultural epidemiological studies of dementia. International Psychogeriatrics.

[ref-22] Wattmo C, Jedenius E, Blennow K, Wallin AK (2013). Dose and plasma concentration of galantamine in Alzheimer’s disease—clinical application. Alzheimer’s Research & Therapy.

[ref-23] Wilcock GK, Lilienfeld S, Gaens E (2000). Efficacy and safety of galantamine in patients with mild to moderate Alzheimer’s disease: multicentre randomised controlled trial. Galantamine International-1 Study Group. BMJ.

[ref-24] Wilkinson D, Murray J (2001). Galantamine: a randomized, double-blind, dose comparison in patients with Alzheimer’s disease. International Journal of Geriatric Psychiatry.

[ref-25] Yang YH, Chen CH, Chou MC, Li CH, Liu CK, Chen SH (2013). Concentration of donepezil to the cognitive response in Alzheimer disease. Journal of Clinical Psychopharmacology.

[ref-26] Zhao Q, Brett M, Van Osselaer N, Huang F, Raoult A, Van Peer A, Verhaeghe T, Hust R (2002). Galantamine pharmacokinetics, safety, and tolerability profiles are similar in healthy Caucasian and Japanese subjects. Journal of Clinical Pharmacology.

